# Retinal debris triggers cytotoxic damage in cocultivated primary porcine RPE cells

**DOI:** 10.3389/fnins.2024.1401571

**Published:** 2024-07-24

**Authors:** Natalie Wagner, Teresa Tsai, Sabrina Reinehr, Janine Theile, H. Burkhard Dick, Stephanie C. Joachim

**Affiliations:** Experimental Eye Research Institute, University Eye Hospital, Ruhr-University Bochum, Bochum, Germany

**Keywords:** age-related macular degeneration, coculture system, deposits, neuroretina, retinal pigment epithelium (RPE)

## Abstract

**Introduction:**

One of the most common causes of vision loss in the elderly population worldwide is age-related macular degeneration (AMD). Subsequently, the number of people affected by AMD is estimated to reach approximately 288 million by the year 2040. The aim of this study was to develop an *ex vivo* model that simulates various aspects of the complex AMD pathogenesis.

**Methods:**

For this purpose, primary porcine retinal pigment epithelial cells (ppRPE) were isolated and cultured. One group was exposed to medium containing sodium iodate (NaIO_3_) to induce degeneration. The others were exposed to different supplemented media, such as bovine serum albumin (BSA), homogenized porcine retinas (HPR), or rod outer segments (ROOS) for eight days to promote retinal deposits. Then, these ppRPE cells were cocultured with porcine neuroretina explants for another eight days. To assess the viability of ppRPE cells, live/dead assay was performed at the end of the study. The positive RPE65 and ZO1 area was evaluated by immunocytochemistry and the expression of *RLBP1*, *RPE65*, and *TJP1* was analyzed by RT-qPCR. Additionally, drusen (*APOE*), inflammation (*ITGAM*, *IL6*, *IL8*, *NLRP3*, *TNF*), oxidative stress (*NFE2L2*, *SOD1*, *SOD2*), and hypoxia (*HIF1A*) markers were investigated. The concentration of the inflammatory cytokines IL-6 and IL-8 was determined in medium supernatants from day 16 and 24 via ELISA.

**Results:**

Live/dead assay suggests that especially exposure to NaIO_3_ and HPR induced damage to ppRPE cells, leading in a significant ppRPE cell loss. All supplemented media resulted in decreased RPE-characteristic markers (RPE65; ZO-1) and gene expression like *RLBP1* and *RPE65* in the cultured ppRPE cells. Besides, some inflammatory, oxidative as well as hypoxic stress markers were altered in ppRPE cells cultivated with NaIO_3_. The application of HPR induced an enhanced *APOE* expression. Pre-exposure of the ppRPE cells led to a diminished number of cones in all supplemented media groups compared to controls.

**Discussion:**

Overall, this novel coculture model represents an interesting initial approach to incorporating deposits into coculture to mimic AMD pathogenesis. Nevertheless, the effects of the media used need to be investigated in further studies.

## 1 Introduction

Age-related macular degeneration (AMD) is a main cause of untreatable severe visual impairment or even blindness worldwide. In industrialized countries, it is the most common eye disease in people over the age of 55. It is predicted to affect 288 million individuals worldwide by 2040 (World Health Organization, 2005; Wong et al., 2014). AMD pathogenesis is associated with changes in the homeostasis of photoreceptor cells, retinal pigment epithelium (RPE), and Bruch’s membrane (Somasundaran et al., 2020). Another hallmark is the development of drusen and diffuse deposits within the retina. Especially drusen formation has been broadly investigated and many drusen components are already identified via, for example, proteomics (Mullins et al., 2000; Crabb et al., 2002; Wang et al., 2010), but due to its complexity some points are still unknown. The major driving force of drusen formation, on a molecular base, is poorly understood yet. Crabb et al. (2002) performed detailed proteomic analysis of human drusen samples and provided initial insights pointing to proteins of specific groups that seem to be involved in drusenogenesis. They identified 129 proteins, including metalloproteinase inhibitor 3, clusterin, vitronectin, vimentin, serum albumin, crystalline, and complement components. The mentioned proteins were increased in AMD donors (Crabb et al., 2002; Crabb, 2014). Some of those components are considered to originate from the neural side, meaning RPE or photoreceptors, while others are assumed to have a systemic origin, in particular from Bruch’s membrane, choroid, or blood (Penfold et al., 2001; Curcio et al., 2011; Curcio and Johnson, 2013; Curcio, 2018). Furthermore, Wang et al. (2010) showed that human fetal RPE cells secrete deposits associated with drusen proteins, which undermines the hypothesis that the RPE itself is a potential source of drusen components. The results of Pilgrim et al. (2017) further support the assumption that drusen formation originates from the RPE cells, as they were able to that show the formation of sub-RPE deposits in primary porcine RPE (ppRPE) cells is initiated by the RPE itself. These deposits consisted of hydroxyapatite and remarkably showed a striking resemblance to human drusen (Pilgrim et al., 2017). However, there are other research groups considering blood plasma as a significant source of drusen proteins (Curcio, 2018). Johnson et al. (2011) presented a novel human fetal RPE model that displayed potential to mimic some key aspects of the early stages of AMD. They demonstrated the accumulation of sub-RPE deposits containing molecular components of human drusen, but also illuminated the role of the complement system. In addition, these sub-RPE deposits were found to be rich in apolipoprotein E (APOE) (Johnson et al., 2011).

Recently, exposure to various deposit-inducing media from supplemented protein-rich sera, retinal extracts, or photoreceptor outer segments were investigated with respect to the formation of sub-RPE deposits. Amin et al. (2004) examined primary RPE cells exposed to three different media, highly enriched with either bovine serum albumin (BSA), isolated rod outer segments (ROOS), or homogenized porcine retina (HPR) to induce deposit formation. The mentioned media should promote and accelerate the formation of deposits. In their study, only ppRPE cells exposed to HPR-enriched medium for eight days showed the ability to form deposits.

In AMD, two late stages are distinguished, defined as “wet” (exudative) and “dry” (geographic atrophy). The angiogenesis and increased vascular permeability observed in wet AMD is mediated in part by an upregulation of the vascular endothelial growth factor (VEGF) (Green and Enger, 1993; Spaide and Curcio, 2010; Ambati and Fowler, 2012; Ferris et al., 2013). Despite the evolving state of research, the therapeutic options for AMD are still very limited and at present, mostly address the late stage of wet AMD. The gold standard procedure includes regular costly injections of VEGF inhibitors. Consequently, there is a certain need for adequate treatment options, especially for dry AMD. For this form, no approved drugs are currently available in Europe. In 2023, pegcetacoplan was approved by the Food and Drug Administration for use in the USA to slow the rate of geographic atrophy (Liao et al., 2020; Nadeem et al., 2023).

Given the ethical reservations about human material or animal testing, the homologous porcine eye, which can be obtained as a waste byproduct of the meat industry, represents a good alternative source in research. Due to the extremely complex pathogenesis of AMD, it is difficult to find suitable *in vitro* models. Since many different structures of the eye and mechanisms are involved in AMD, it is very challenging to reproduce them in culture.

In previous studies of our group, a gentle explantation technique for the neuroretina has been established, enabling the investigation of rod and cones *in vitro* (Wagner et al., 2020). Based on those findings, our *ex vivo* model should be further enhanced. Hence, we wanted to combine our neuroretina cultivation technique with various pretreated ppRPE feeder layers adapted from the approaches described by Amin et al. (2004) and Pilgrim et al. (2017) in order to achieve a coculture model with drusen-like deposits. This will enable us to examine both the molecular and cellular aspects of the development of RPE deposits and their effects on neuroretina explants in order to draw conclusions about AMD pathogenesis.

## 2 Materials and methods

### 2.1 Preparation of supplemented media

In this project, four different media supplements were tested on ppRPE cells. These cells were subsequently cocultured with porcine neuroretina. All media were freshly prepared immediately before use, while supplements were prepared beforehand.

In the degeneration control group, cells received a serum-free ppRPE medium supplemented with 3 mM sodium iodate (NaIO_3_, Sigma-Aldrich. St. Louis, MA, USA).

In the BSA group, cells received ppRPE medium supplemented with 0.42 w/V BSA (Sigma-Aldrich) and 10% fetal calf serum (FCS) (Amin et al., 2004).

In the HPR group, freshly collected porcine retinas were homogenized by extensive trituration and then frozen in a penicillin-streptomycin solution (Sigma-Aldrich) with liquid nitrogen and stored at −80°C until use. Then, 20 ml serum-free ppRPE medium was supplemented with one homogenized porcine retina (Amin et al., 2004).

For the ROOS group, retina tissue from freshly enucleated porcine was used. The procedure was previously described by Klettner et al. (2011) and originally adapted from Schraermeyer et al. (1997). In brief, porcine retina was homogenized in specific homogenization buffer (34% Sucrose), 65 mM NaCl, 2 mM MgCl_2_, and 5 mM HEPES (all: Sigma-Aldrich) followed by a centrifugation step at 3,800 rpm for 4 min. The supernatant was diluted in 10 mM HEPES solution. Then, another centrifugation step at 3,800 rpm was performed for 5 min resulting in the isolation of ROOS in the supernatant. Isolated ROOS were stored at −80°C until further use. Then, 6 ml serum-free ppRPE medium was supplemented with 100 μl isolated ROOS (Schraermeyer et al., 1997).

### 2.2 Isolation of ppRPE cells

Freshly collected porcine eyes were used for isolating ppRPE cells. These were obtained from a nearby abattoir, where they were otherwise discarded as a waste byproduct of meat production. The eyes were transported to the laboratory on ice in an antiseptic solution (Videne antiseptic solution; Mundipharma GmbH, Cambridge, UK), which was diluted 1:5 in phosphate-buffered saline (PBS; Sigma-Aldrich). For further processing of the specimens, excessive tissue was removed from the eyes with scissors. Then, the eyes were briefly dipped in ethanol and stored in PBS on ice until dissection. Under sterile conditions, the eyes were now opened performing an incision into the cornea. The anterior part was discarded. Each eye cup was placed in one well of a 12-well plate (Sarstedt, Nümbrecht, Germany). Afterwards, every eye cup was incubated with 1 ml of 1 mM ethylenediaminetetraacetic (EDTA, Sigma-Aldrich) at 37°C for 10 min. The neuroretina was carefully removed from the underlying RPE. Then, all eye cups were filled and incubated in 1 mL of 1× Trypsin-EDTA at 37°C for 45 min. Next, the RPE cells were collected by gentle trituration. The ppRPE cells were centrifuged at 3,000 rpm for 10 min before being transferred into T25 flasks (Sarstedt) in 5 ml ppRPE growth medium (DMEM/F12, Gibco Life Technologies, Carlsbad, CA, USA) supplemented with 10% fetal bovine serum superior (Sigma-Aldrich), 2% penicillin/streptomycin (Sigma-Aldrich), 2% B27 (Gibco Life Technologies), and 1% gentamicin (Sigma-Aldrich). Then, ppRPE were cultured for 8 days (day 0–8), medium was replaced completely at day 4.

### 2.3 Cultivation of ppRPE cells

**FIGURE 1:**
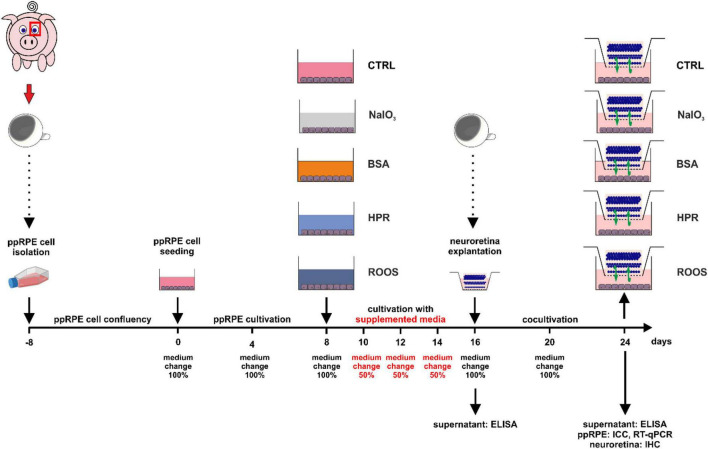
Illustration of the experimental set-up and timeline. Fresh eyes were used to isolate ppRPE cells and these cells were cultivated in ppRPE medium (DMEM/F12) in T25 flasks until they reached confluence (days –8 to 0). Subsequently, about 300,000 cells were seeded per well in 12-well plates and allowed to grow in ppRPE medium for eight days (days 0 to 8). At day 8, the supplemented media were applied: NaIO_3_, BSA, HPR, or ROOS. Control samples received no supplement in their ppRPE medium (CTRL). The media were applied for eight days and every two days, 50% of the supplemented medium was replaced (days 8 to 16). From day 16 on, a coculture was performed by adding freshly isolated porcine neuroretina explants to all wells (days 16 to 24). Also, the supplemented media were replaced with 1:1 ppRPE and neuroretina (Neurobasal-A) medium. Medium was exchanged at day 20. On days 16 and 24, ELISA analysis of the medium supernatant was performed. On day 24, immunocytochemistry (ICC) and RT-qPCR were conducted of the ppRPE cells and immunohistochemistry (IHC) from the neuroretina samples.

The ppRPE cells in each flask were dissociated with 1 ml Trypsin-EDTA at 37°C for 5 min. First, 300,000 ppRPE cells were seeded per collagen I precoated well (Gibco, Thermo Fisher Scientific, Waltham, USA) of a 12-well plate. The ppRPE cells were then cultured in ppRPE medium at 37°C and 5% CO_2_ for 8 days to create a monolayer (days 0–8, Figure 1). The medium was replaced on day 4.

### 2.4 Application of supplemented media to ppRPE cells

At day 8, the application of the supplemented media was started. In this study, we investigated three different debris induction media as well as a degeneration group and a control group (Figure 1). Hence, five groups were compared in this study:

1. CTRL = control group (serum-free ppRPE medium without additional treatment)
2. NaIO_3_ = degeneration control group (serum-free ppRPE medium supplemented with 3 mM NaIO_3_)
3. BSA (serum-free ppRPE medium supplemented with 0.42 w/V BSA and 10% FCS)
4. HPR (20 ml serum-free ppRPE medium supplemented with one homogenized porcine retina)
5. ROOS (6 ml serum-free ppRPE medium supplemented with 100 μl isolated ROOS)

All media were freshly prepared immediately before use. During the deposit-induction period of 8 days, 50% of the media was changed every second day.

### 2.5 Preparation of porcine neuroretina explants and initiation of coculture

At day 16, neuroretina explants from porcine eyes were obtained using the so-called forceps technique as previously described (Wagner et al., 2020). In brief, a skin punch (Ø = 6 mm, Pfm medical AG, Cologne, Germany) was used to punch out two neuroretina explants from the visual strip of each porcine eye under aseptic conditions. Using forceps, the explant was lifted at the sclera, flipped and placed, photoreceptors facing up, on a cell culture insert (Millipore, Burlington, MA, USA).

For the coculture, the retinal explants were cultured with ppRPE cells (from 2.4) in 1 ml of 1:1 neuroretina medium and ppRPE medium at 37°C and 5% CO_2_ for 8 days (days 16–24, Figure 1). The neuroretina medium was composed of Neurobasal-A medium (Life Technologies) supplemented with 0.8 mM L-glutamine, 2% B27, 1% N2 (all: Life Technologies), and 2% penicillin/streptomycin (Sigma-Aldrich). Medium was replaced at day 20.

### 2.6 Enzyme-linked immunosorbent assays

Medium supernatant from all groups was collected at day 16 and 24 (*n* = 6 samples/group/time point) (Klettner et al., 2021). The levels of the inflammatory cytokines interleukin (IL)-6 (ab100755, Abcam, Cambridge, UK) and IL-8 (ab113352, Abcam) were analyzed in those supernatant samples via multiplex enzyme-linked immunosorbent assay (ELISA). Colorimetric solid phase sandwich ELISA were performed according to the manufacturer’s instructions using a Microplate Reader (AESKU Reader; AESKU.DIAGNOSTICS, Wendelsheim, Germany).

### 2.7 Live/dead cell staining

To evaluate cell viability, a live/dead double staining kit with calcein AM and propidium iodide was used (QIA76, Sigma-Aldrich). The procedure was performed according to the manufacturer’s standard protocol (*n* = 6 samples/group).

### 2.8 Immunocytochemistry staining (ICC) of ppRPE cells

The RPE-specific protein RPE65 and the zonula occludens marker ZO-1 were labeled in ppRPE cells (*n* = 6 samples/group). In brief, ppRPE cells were fixed with 4% paraformaldehyde (PFA; Merck, Darmstadt, Germany) at room temperature for 10 min. Thereafter, the blocking-buffer solution containing 10% goat and/or donkey serum (according to the used secondary antibody), 1% BSA, and 0.2% Triton X in PBS, was applied for 1 h. The ppRPE cells were incubated with the primary antibody (Table 1) diluted in blocking buffer solution at 4°C overnight. On the following day, ppRPE cells were incubated with corresponding secondary antibodies (Table 1) diluted in blocking-buffer solution for 1 h. Cell nuclei were stained with 4′,6 diamidino-2-phenylindole (DAPI, Serva Electrophoresis, Heidelberg, Germany) for 5 min.

**TABLE 1:** List of primary and corresponding secondary antibodies used in this study.

Primary antibodies				Secondary antibodies			
Antibody	Catalog number	Company	Dilution	Antibody	Catalog number	Company	Dilution
Anti-IL-6	21865-1-AP	Proteintech	1:200	Donkey anti-rabbit Alexa Fluor 488	711-547-003	Jackson Immuno Research	1:500
Anti-IL-8	M801	Invitrogen	1:200	Donkey anti-mouse Alexa Flour 555	ab150106	Abcam	1:500
Anti-M/L-opsin	AB5405	Millipore	1:1200	Donkey anti-rabbit Alexa Fluor 488	711-547-003	Jackson Immuno Research	1:600
Anti-rhodopsin	ab3267	Abcam	1:400	Goat anti-mouse Alexa Fluor 488	A-11029	Invitrogen	1:500
Anti-RPE65	ab13826	Abcam	1:200	Donkey anti-mouse Alexa Fluor 555	ab150106	Abcam	1:500
Anti-ZO-1	61-7300	Invitrogen	1:100	Goat anti-rabbit Alexa Fluor 488	A-11008	Invitrogen	1:500

### 2.9 Immunohistochemistry staining (IHC) of neuroretina samples

Neuroretina explants were fixed with 4% PFA for 15 min. Then, explants were drained with 15% sucrose solution (Sigma-Aldrich) for 15 min and 30% sucrose solution for 30 min. Finally, samples were embedded in NEG-50™ Tissue Tek medium (Thermo Fisher Scientific) and stored at −80°C. The embedded neuroretina samples were later cut into 10 μm cross-sections using a microtome HM 550 Kryostat (Thermo Fisher Scientific). Three sections were placed per HistoBond^®^ microscope slide (Paul Marienfeld GmbH & Co. KG, Lauda-Königshofen, Germany). The slides were air-dried at room temperature overnight, then fixed in ice-cold acetone for 10 min, and stored at −20°C until further processed.

Specific primary antibodies were used for IHC to detect rod (rhodopsin) and cone (M/L-opsin) photoreceptors as well as the cytokines IL-6 and IL-8 of the retina (*n* = 4–5 samples/group, 6 sections/sample; Table 1) (Kuehn et al., 2016; Hurst et al., 2017). Neuroretina sections were defrosted at 37°C for 15 min. Following, the slides were rinsed 5 min in PBS and blocked 1 h with sera (10% goat or donkey) diluted in 0.1 or 0.3% Triton X-100+PBS and 1% BSA. Next, the neuroretina sections were incubated with primary antibodies diluted in blocking solution at room temperature overnight. On the next day, the slides were washed in PBS and then incubated with corresponding secondary antibodies labeled with Alexa Fluor 488 or Alexa Fluor 555 at room temperature for 1 h. 0.01 μg/mL DAPI was used to label cell nuclei. In one sample, the primary antibody was omitted to serve as a no primary antibody control. Finally, all neuroretina sections were covered in Shandon mount media (Thermo Fisher Scientific).

To visualize apoptotic cells, a terminal deoxynucleotidyl transferase-mediated dUTP nick end labeling (TUNEL) assay was utilized (*n* = 5 samples/group, 3 sections/sample). This assay was performed according to the manufacturer’s instructions (TMR red, 12156792910, Roche^®^, Basel, Switzerland).

Also, a DAPI (0.01 μg/mL) staining of cell nuclei (*n* = 4–5 samples/group, 3 sections/sample) was performed to evaluate the positive staining area in the outer nuclear layer (ONL) after coculture. Hence, possible degeneration of the ONL could be determined by measuring the DAPI^+^ area.

### 2.10 Evaluation of ICC and IHC staining

Exemplary brightfield images of ppRPE cells were taken with a Axio Imager M1 microscope (Zeiss, Oberkochen, Germany). Images of stained ppRPE cells and neuroretina cross-sections were taken via Axio Imager M2 microscope (Zeiss). For ppRPE staining, three images per well were taken. For neuroretina staining, four images per section were taken.

For the evaluation of RPE65 and ZO-1 in ppRPE cells, images were cut into predefined cutouts (300 × 300 pixel). Neuroretina images were cut into cutouts of 800 × 600 pixel (DAPI: 700 × 350 pixel). Afterwards, cell counts or area measurements were performed.

Cell bodies positively labeled with live/dead cell staining assay, M/L-opsin, and TUNEL were counted using the ImageJ (NIH, Bethesda, MD, USA) plugin “cell counter.”

For all other stains (IL-6, IL-8, RPE65, ZO-1, rhodopsin, and DAPI), an area measurement was performed using an established ImageJ macro (Reinehr et al., 2018; Benning et al., 2020; Wagner et al., 2020). Hence, original images were transferred into grayscale (32-bit) and then the background was subtracted (Table 2). For each image, a lower and upper threshold was defined. During the further analyses, the mean value for the lower and upper threshold was used (Table 2).

**TABLE 2:** Background subtraction as well as lower and upper thresholds used for staining analysis using ImageJ software.

Staining	Background subtraction	Lower threshold	Upper threshold
DAPI	50	19.31	83.99
IL-6	50	10.20	233.48
IL-8	50	6.91	234.85
Rhodopsin	50	14.65	123.00
RPE-65	72	15.40	112.41
ZO-1	68	14.85	103.85

### 2.11 Quantitative real-time PCR (RT-qPCR) of ppRPE cells

After 24 days of cultivation, RNA of ppRPE pellets was extracted according to the manufacturer’s instructions of the GenElute™ Mammalian Total RNA Miniprep Kit (RTN350-1KT, Sigma-Aldrich) (Reinehr et al., 2019, 2021; Weiss et al., 2021). To measure the quantity and quality of the extracted RNA a NanoDrop ONE (Thermo Fisher Scientific) was utilized. For synthesis of cDNA, 1 μg RNA was used and transcribed with First Strand cDNA Synthesis Kit (K1612, Thermo Fisher Scientific). RT-qPCR analysis was performed via DyNAmo ColorFlash SYBR Green qPCR-Kit (F416L, Thermo Fisher Scientific) on the PikoReal RT-qPCR Cycler (Thermo Fisher Scientific). All used primers and gene sequences were listed in Table 3. *RPE65*, *RLBP1*, and *TJP1* expression as well as inflammation markers (*ITGAM*, *IL6*, *IL8*, *NLRP3*, *TNF*), oxidative stress markers (*NFE2L2*, *SOD1*, *SOD2*), a hypoxia marker (*HIF1A*), an extracellular matrix (ECM) remodeling marker (*MMP2*), and additionally a drusen marker (*APOE*) were investigated via RT-qPCR (*n* = 6/group). Data were normalized to housekeeping genes *ACTIN* (*ACTB*) as well as *HISTON H3* (*H3-3A*) and evaluated using REST© software (Qiagen, Hilden, Germany).

**TABLE 3:** List of all RT-qPCR primer pairs used for analyses.

Gene	Oligonucleotides 5′ 3′	GenBank (accession number)	Basepairs
*ACTB* for	CTCTTCCAGCCTTCCTTC	XM_021086047.1	178
*ACTB* rev	GGGCAGTGATCTCTTTCT		
*H3-3A* for	ACTGGCTACAAAAGCCGCTC	NM_213930.1	232
*H3-3A* rev	ACTTGCCTCCTGCAAAGCAC		
*APOE* for	GTGGGTTGCTTTGGTGGTAAC	NM_214308	152
*APOE* rev	GCAGGTAATCCCAGAAGCGG		
*ITGAM* for	AGAAGGAGACACCCAGAGCA	XM_021086380.1	169
*ITGAM* rev	GTAGGACAATGGGCGTCACT		
*HIF1A* for	ACTTCTGGGCCGCTCAATTT	NM_001123124.1	133
*HIF1A* rev	TCCACCTCTTTTGGCAAGCA		
*IL6* for	GCAGTCACAGAACGAGTGGA	NM_214399.1	84
*IL6* rev	CTCAGGCTGAACTGCAGGAA		
*IL8* for	*TTCCAAACTGGCTGTTGCCT*	M86923.1	178
*IL8* rev	*ACAGTGGGGTCCACTCTCAA*		
*MMP2* for	*GCAGTGATGGCAAGTTGTGG*	NM_214192	214
*MMP2* rev	*TTGACATCGTCGTGGGACAG*		
*NFE2L2* for	GCCGACTATTCCCAGGTAGC	XM_003133500.6	713
*NFE2L2* rev	GTTGTGCTTTCACGGTGGTC		
*NLRP3* for	GTGAGCAAGCCTTCCAGGAT	NM_001256770	105
*NLRP3* rev	CACCTTCTGCCAGTTTGTGC		
*RLBP1* for	CCACTTCATCCACCAGCCAT	XM_003356660.4	92
*RLBP1* rev	GGACAAAGACCCTCTGGAGC		
*RPE65* for	ACGTACGGGCAATGACTGAG	XM_003127931.5	438
*RPE65* rev	ATCGGTCACTGCAGGGGAAT		
*SOD1* for	AAAACATGGTGGGCCAAAGG	NM_001190422.1	72
*SOD1* ref	CCATCTTTGCCAGCAGTCAC		
*SOD2 for*	*CAGCTCGAGCAGGAATCTGG*	NM_214127.2	87
*SOD2* rev	*CCATAGTCGTACGGCAGGTC*		
*TJP1* for	TCAAGGTCTGCCGAGACAAC	XM_003480423.4	122
*TJP1* rev	CAGCTCCACGGGCTTCAG		
*TNF for*	CCACCAACGTTTTCCTCACT	JF831365.1	296
*TNF* rev	CCAAAATAGACCTGCCCAGA		

For relative quantification of mRNA levels, two house-keeping genes, *β-ACTIN* (*ACTB*) and *H3.3 HISTONE A* (*H3-3A*), were used for the normalization.

### 2.12 Statistical evaluation

ANOVA followed by Tukey post-hoc test (ICC, IHC, ELISA) was performed using Statistica Software (Version 14.1, Dell, Tulsa, OK, USA). ELISA levels between day 16 and day 24 for each group were compared by Student’s *t*-test (Statistica). Regarding RT-qPCR, the relative expression values were assessed via Pair Wise Fixed Reallocation Randomisation Test using REST^©^ software (Pfaffl, 2001; Reinehr et al., 2019; Mueller-Buehl et al., 2021).

ICC and IHC data are shown as mean ± SEM and ELISA as mean ± SEM ± SD. RT-qPCR data is displayed as mean ± quartile ± minimum/maximum. *P*-values were considered statistically significant when *p* < 0.050. The following *p*-values are displayed: *p < 0.050, ^**^p < 0.010, and ^***^p < 0.001 vs. CTRL; ^#^*p* < 0.050, ^##^*p* < 0.010, ^###^*p* < 0.001 vs. NaIO_3_; ^$$^*p* < 0.010; ^$$$^*p* < 0.001 vs. BSA; ^¥¥^*p* < 0.010 vs. HPR; and ^••^*p* < 0.010, ^•••^*p* < 0.001 vs. ROOS.

## 3 Results

### 3.1 Supplemented media leads to significant ppRPE loss

**FIGURE 2:**
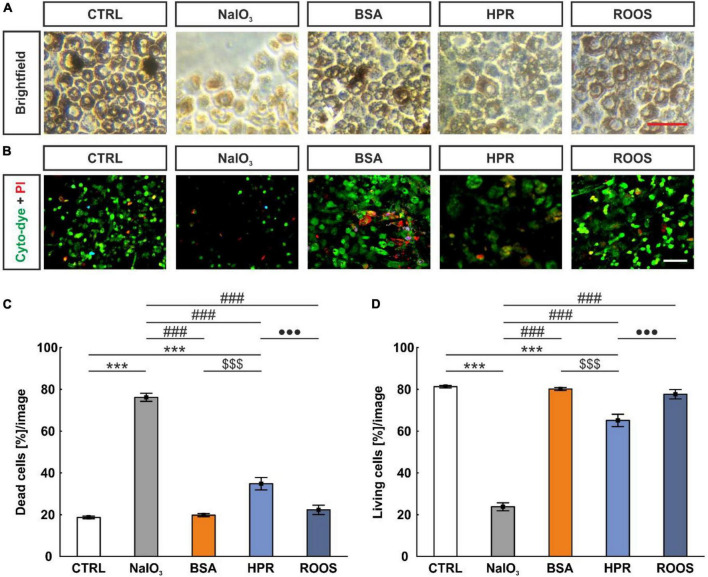
NaIO_3_ exposure led to enhanced ppRPE cell death. **(A)** To evaluate the effects of different media on the survival of ppRPE cells brightfield images were obtained. **(B)** Also, cyto-dye (green) and PI (red) stain was performed. **(C)** The NaIO_3_ and HPR exposed ppRPE cells showed significantly more dead cells. **(D)** Accordingly, living cells per section were significantly diminished in the NaIO_3_ as well as in the HPR group. CTRL, control; BSA, bovine serum albumin; HPR, homogenized porcine retina; ROOS, rod outer segments. Scale bars: brightfield: 50 μm, ICC: 20 μm, values are mean ± SEM, *n* = 6/group, ***p < 0.001 vs. CTRL; ^###^*p* < 0.001 vs. NaIO_3_; ^$$$^*p* < 0.001 vs. BSA; ^•••^*p* < 0.001 vs. ROOS.

Brightfield images of ppRPE from all five groups showed identical, characteristically dark pigmentation and cobblestone morphology (Figure 2A). To investigate cell viability of ppRPE cells after application of retinal supplemented media, live and dead cell staining was performed (Figure 2B).

A reduction of the total cell number was observed in all supplemented groups (NaIO_3_: 10.83 ± 0.99 cells, BSA: 106.56 ± 1.94 cells, HPR: 76.22 ± 2.16 cells, ROOS: 95.28 ± 3.80 cells) in contrast to CTRL ppRPE cells (128.06 ± 2.26 cells; all *p* < 0.001). The lowest number of ppRPE cells was detected in the NaIO_3_ samples also compared to all other groups (all: *p* < 0.001; data not shown).

In addition, the number of dead or living cells was evaluated. This analysis showed a significantly higher percentage of dead cells in the NaIO_3_ group (76.19 ± 1.91 cells [%]/image) in contrast to all other groups (CTRL: 18.71 ± 0.64 cells [%]/image, BSA: 19.80 ± 0.75 cells [%]/image, HPR: 34.87 ± 2.96 cells [%]/image, ROOS: 22.33 ± 2.24 cells [%]/image; all: *p* < 0.001). Further, the HPR group showed significantly more dead cells compared to CTRL, BSA, and ROOS samples (all: *p* < 0.001; Figure 2C).

Accordingly, the lowest percentage of living cells after media treatment was observed in NaIO_3_ ppRPE cells (23.81 ± 1.91 cells [%]/image) compared to all other specimens (CTRL: 81.29 ± 0.64 cells [%]/image, BSA: 80.20 ± 0.75 cells [%]/image, HPR: 65.13 ± 2.96 cells [%]/image, ROOS: 77.67 ± 2.24 cells [%]/image; all: *p* < 0.001). Thus, fewer living cells were observed in the HPR group when compared to CTRL, BSA, and ROOS specimens (all: *p* < 0.001; Figure 2D).

### 3.2 Exposure to supplemented media reduces expression of RPE-specific markers

**FIGURE 3:**
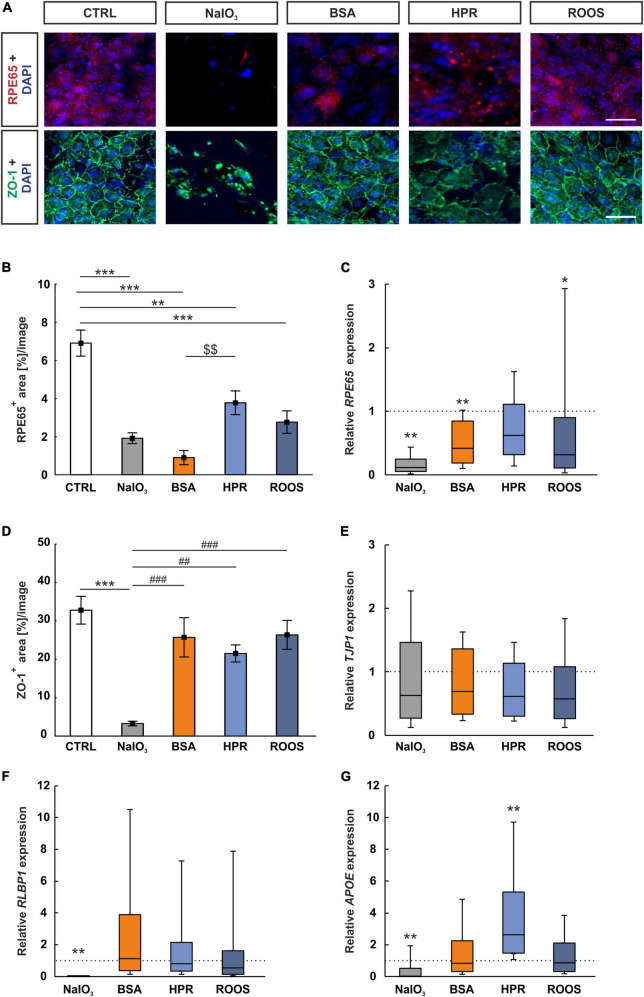
RPE65 decreased in all supplemented groups. **(A)** Immunocytochemistry images were evaluated to assess the RPE-specific RPE65 (red) and the distribution of the tight junction marker ZO-1 (green). Cell nuclei were stained with DAPI (blue). **(B)** RPE65^+^ stained area was significantly diminished in all four supplemented groups, while BSA ppRPE cells showed the lowest values compared to CTRL. **(C)** Relative expression of *RPE65* was significantly reduced in NaIO_3_, BSA, and ROOS exposed ppRPE cells in comparison to CTRLs. **(D)** ZO-1^+^ area was significantly decreased in the NaIO_3_ group. **(E)** *TJP1* expression was comparable in all groups. **(F)** The RPE-specific gene *RLBP1* was significantly downregulated in RPE cells exposed to NaIO_3_, while all other groups did not show significant differences to CTRLs. **(G)** *APOE* expression was significantly downregulated in the NaIO_3_ group and compared to all other group enhanced in the HPR group when compared to CTRL. CTRL, control; BSA, bovine serum albumin; HPR, homogenized porcine retina; ROOS, rod outer segments. Scale bars: 20 μm, values in B+D are mean ± SEM and in **(C,E,F,G)** median ± quartile ± minimum/maximum, the dotted lines in **(C,E,F,G)** represent the relative expression of the CTRL group, *n* = 6/group, *p < 0.050, **p < 0.010, ***p < 0.001 vs. CTRL; ^$$^*p* < 0.01 vs. BSA. ^##^*p* < 0.010, ^###^*p* < 0.001 vs. NaIO_3_.

RPE65 is a protein abundant in ppRPE cells. An anti-RPE65 antibody determined uniformly distributed RPE65 protein over the ppRPE cell monolayer (Figure 3A). RPE65^+^ stained area was significantly smaller in all four supplemented groups, namely NaIO_3_ (1.92 ± 0.28 area[%]/image; *p* < 0.001), BSA (0.90 ± 0.37 area[%]/image; *p* < 0.001), HPR (3.78 ± 0.62 area[%]/image; *p* = 0.003), and ROOS (2.76 ± 0.59 area[%]/image; *p* < 0.001) in comparison to the CTRL group (6.91 ± 0.68 area[%]/image). The RPE65^+^ stained area was significantly higher in the HPR supplemented group compared to the BSA one (*p* = 0.007; Figure 3B).

The *RPE65* mRNA expression was examined by RT-qPCR. Relative *RPE65* expression was significantly diminished in NaIO_3_ ppRPE cells in contrast to CTRL ppRPE cells (0.12-fold expression; *p* = 0.001). Moreover, a significant downregulation could also be observed in BSA ppRPE cells compared to CTRL ones (0.42-fold expression; *p* = 0.005). While no changes were noted in the *RPE65* mRNA levels in the HPR group (0.62-fold expression; *p* = 0.131), significantly downregulated mRNA levels were noted in ROOS ppRPE cells (0.32-fold expression; *p* = 0.049; Figure 3C).

The condition of the tight junctions (ZO-1) in cocultured ppRPE cells was investigated. Each ppRPE cell was outlined by ZO-1^+^ area (Figure 3A). A significantly decreased ZO-1^+^ area was detected in ppRPE cells, which were exposed to NaIO_3_ (3.26 ± 0.61 area[%]/image; *p* < 0.001) in contrast to CTRL ppRPE cells (32.79 ± 3.62 area[%]/image). A decreased ZO-1^+^ area was also noted in NaIO_3_ samples compared to the BSA (25.72 ± 5.12 area[%]/image; *p* < 0.001), the HPR (21.52 ± 2.22 area[%]/image; *p* = 0.007), as well as the ROOS group (26.36 ± 3.75 area[%]/image; *p* < 0.001). No differences in the ZO-1^+^ area were revealed in BSA, HPR, and ROOS treated ppRPE cells compared to CTRL (all: *p* > 0.050; Figure 3D).

The tight junction marker ZO-1 is also known as tight junction protein-1 (*TJP1*) and was used as a corresponding RT-qPCR marker. The mRNA level of *TJP1* in the four supplemented groups was comparable to the CTRL level (NaIO_3_: 0.63-fold expression, BSA: 0.69-fold expression, HPR: 0.61-fold expression; ROOS: 0.57-fold expression; all: *p* > 0.050; Figure 3E).

Expression of the RPE-specific cellular retinaldehyde-binding protein (CRALBP), which is transcribed from the *RLBP1* gene (Lima de Carvalho et al., 2020), was also investigated by RT-qPCR analysis. The *RLPB1* expression was significantly diminished in ppRPE cells exposed to NaIO_3_ (0.01-fold expression; *p* = 0.001) in comparison to the CTRL situation. In contrast, expression of *RLPB1* in ppRPE cells of the other supplemented groups was not altered (BSA: 1.13-fold expression, HPR: 0.79-fold expression, ROOS: 0.54-fold expression; all: *p* > 0.050; Figure 3F).

### 3.3 Medium supplemented with homogenized retina promotes APOE expression

Drusen consist of multiple components, including lipids, polysaccharides, and glycosaminoglycans, and different proteins (Crabb et al., 2002). One of these components are apolipoproteins (Amin et al., 2004). Hence, the expression of *APOE* in ppRPE cells exposed to the various media has been evaluated via RT-qPCR. In the NaIO_3_ group, a significant downregulation was observed (0.04-fold expression; *p* = 0.008). No difference was observed in the BSA supplement group (0.83-fold expression; *p* > 0.050). Notably, ppRPE cells exposed to HPR medium showed significantly enhanced *APOE* expression in contrast to CTRL ppRPE cells (2.64-fold expression; *p* = 0.003). The ROOS group (0.87-fold expression; *p* > 0.050) showed similar *APOE* gene expression levels as CTRLs (Figure 3G).

### 3.4 Supplemented media triggers inflammatory response in ppRPE cells

**FIGURE 4:**
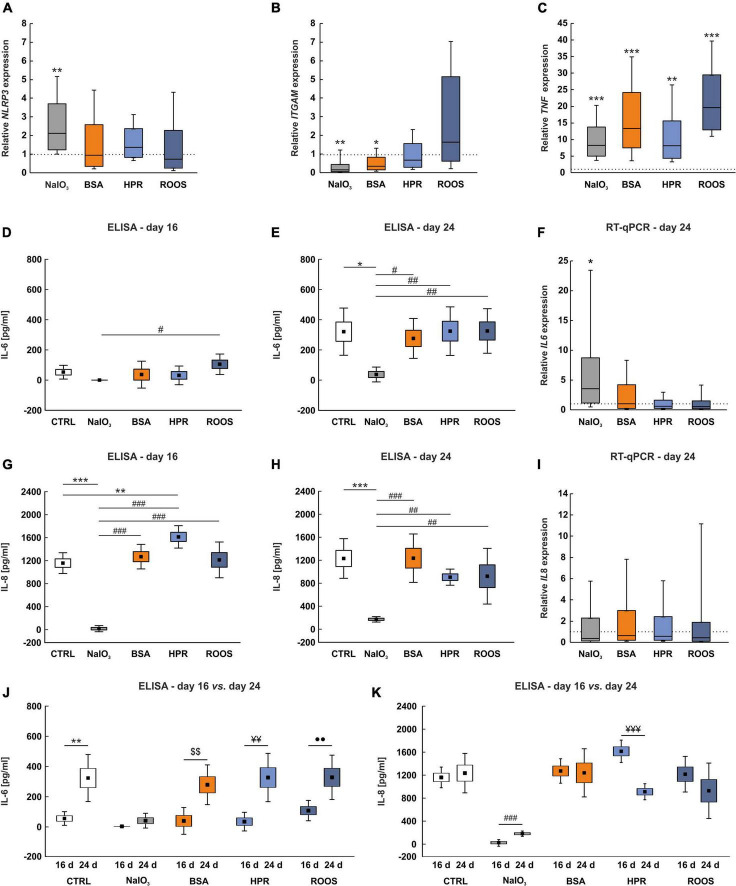
Enhanced *TNF* expression in supplemented groups. **(A)** *NLRP3* inflammasome expression was increased in the NaIO_3_ group, while expression was not altered in the other supplemented groups. **(B)** *ITGAM* mRNA expression levels showed a significantly diminished expression in BSA and NaIO_3_ samples in comparison to CTRL. **(C)** The inflammation marker *TNF* was highly upregulated in all supplemented groups compared to CTRLs. **(D)** No significant differences in IL-6 protein levels in supernatant samples were noted in comparison to CTRLs at day 16. Solely the NaIO_3_ group demonstrated a significantly decreased IL-6 protein level in supernatants compared to ROOS. **(E)** At day 24, IL-6 protein level in supernatant samples was significantly diminished in NaIO_3_ exposed ppRPE cells in comparison to all other groups. **(F)** On the other hand, *IL6* mRNA expression was significantly enhanced in ppRPE cells after NaIO_3_ exposure in contrast to CTRL. **(G)** IL-8 protein level was significantly diminished in supernatant samples of the NaIO_3_ group on day 16, when compared to all other groups. On the other hand, in HPR supernatants the IL-8 level was significantly enhanced in contrast to CTRL. **(H)** At day 24, IL-8 level was significantly reduced in supernatants of the NaIO_3_ group in comparison to all other groups. **(I)** *IL8* mRNA expression was unaltered in ppRPE samples from all groups. **(J)** By comparing ELISA measurements between both points in time for each group, the IL-6 levels were significantly higher at day 24 in CTRL, BSA, HPR, and ROOS samples when compared to IL-6 levels at day 16, while no changes were observed in NaIO_3_ supernatants. **(K)** The IL-8 levels were similar between both points in time in CTRL, BSA, and ROOS samples. IL-8 levels were found increased in the NaIO_3_ group at day 24 when compared to day 16, while less IL-8 was noted in HPR supernatants. CTRL, control; BSA, bovine serum albumin; HPR, homogenized porcine retina; ROOS, rod outer segments, values in **(D,E,G,H,J,K)** are mean ± SEM ± SD and values in **(A,B,C,F,I)** are median ± quartile ± minimum/maximum, the dotted lines in **(A,B,C,F,I)** represent the relative expression of the CTRL group, *n* = 6/group, *p < 0.050, **p < 0.010, ***p < 0.001 vs. CTRL; ^#^*p* < 0.050, ^##^*p* < 0.010, ^###^*p* < 0.001 vs. NaIO_3_; ^$$^*p* < 0.010 vs. BSA; ^¥¥^*p* < 0.010, ^¥¥¥^*p* < 0.001 vs. HPR; ^••^*p* < 0.010 vs. ROOS.

The NLRP3 inflammasome has been related to AMD by several previous studies (Doyle et al., 2012; Kauppinen et al., 2012; Tarallo et al., 2012; Tseng et al., 2013). The expression of *NLRP3* was significantly upregulated in ppRPE cells exposed to NaIO_3_ in comparison to CTRL ppRPE cells (2.13-fold expression; *p* = 0.004). In contrast, the *NLRP3* expression in the BSA (0.95-fold expression), the HPR (1.38-fold expression), as well as the ROOS group (0.73-fold expression) was comparable to CTRLs (all: *p* > 0.050; Figure 4A).

Macrophages circulate as monocytes and invade tissues recruited by local chemokines. In eyes of AMD patients with different disease stages, macrophages were detected in drusen (Penfold et al., 2001). Integrin subunit α M (*ITGAM*) is a reliable marker for macrophages (Larson and Springer, 1990). In comparison to CTRL situation, NaIO_3_ (0.16-fold expression; *p* = 0.005) and BSA exposure (0.36-fold expression; *p* = 0.020) led to a significant reduction of *ITGAM* expression in ppRPE cells. Exposure to HPR and ROOS resulted in no expression alterations compared with CTRL ppRPE cells (HPR: 0.67-fold expression, ROOS: 1.63-fold expression; both: *p* > 0.050; Figure 4B).

### 3.5 NaIO_3_ exposure to ppRPE cells alters cytokine release

Inflammation plays a key role in the pathogenesis of AMD. Several cytokines, such as tumor necrosis factor alpha (TNF-α), IL-6, and IL-8, are linked with angiogenic properties (Cohen et al., 1996; Yoshida et al., 1997; Sainson et al., 2008). Remarkably, the expression of *TNF* showed a significant upregulation in all supplemented groups, more precisely the NaIO_3_ (8.27-fold expression; *p* < 0.001) the BSA (13.35-fold expression; *p* < 0.001), the HPR (8.15-fold expression; *p* = 0.001), and the ROOS group (19.66-fold expression; *p* < 0.001) in comparison to CTRL ppRPE cells (Figure 4C).

Primary RPE cells abundantly secrete cytokines such as IL-6 and IL-8. Therefore, the secretion of IL-6 and IL-8 after exposure was investigated in supernatants obtained from all groups at day 16 and 24 with ELISA assays. At 16 days, the IL-6 protein level was not altered in the NaIO_3_ (under detection level), BSA (36.36 ± 36.36 pg/ml), HPR (31.48 ± 25.29 pg/ml), and ROOS samples (104.98 ± 27.67 pg/ml) compared to CTRL (52.40 ± 18.56 pg/ml; all: *p* > 0.050). The IL-6 level was significantly decreased in the supernatant of the NaIO_3_ group compared to ROOS samples (*p* = 0.044; Figure 4D).

Interestingly, the IL-6 protein levels were in general higher in supernatants at day 24. However, the IL-6 level was significantly lower in supernatants from the NaIO_3_ group (37.95 ± 20.06 pg/ml) than in CTRL (321.63 ± 63.84 pg/ml; *p* = 0.010) as well as compared to BSA (276.88 ± 53.92 pg/ml; *p* = 0.040), HPR (325.06 ± 65.64 pg/ml; *p* = 0.009), or ROOS group supernatants (326.23 ± 60.25 pg/ml; *p* = 0.009; Figure 4E).

In addition, mRNA expression of *IL6* was significantly increased in ppRPE cells exposed to NaIO_3_ (3.57-fold expression; *p* = 0.016) in comparison to CTRLs. On the other hand, ppRPE cells supplemented with BSA (1.04-fold expression), HPR (0.59-fold expression), or ROOS (0.56-fold expression) did not show any changes in *IL6* expression (all: *p* > 0.050; Figure 4F).

The concentration of IL-8 was also significantly diminished in supernatant collected from ppRPE cells exposed to NaIO_3_ (40.50 ± 21.62 pg/ml) in comparison to CTRL (1,169.63 ± 73.02 pg/ml; *p* < 0.001), BSA (1,280.84 ± 86.31 pg/ml; *p* < 0.001), HPR (1,621.47 ± 78.86 pg/ml; *p* < 0.001), or ROOS samples (1,225.08 ± 125.72 pg/ml; *p* < 0.001) at day 16. An increase of IL-8 protein level was noted in HPR samples compared to CTRL (*p* = 0.007; Figure 4G).

At day 24, IL-8 level in the supernatant was still significantly reduced in the NaIO_3_ group (196.00 ± 18.99 pg/ml) in comparison to CTRL (1,243.82 ± 139.35 pg/ml; *p* < 0.001), BSA (1,248.89 ± 170.10 pg/ml; *p* < 0.001), HPR (921.75 ± 56.74 pg/ml; *p* = 0.007), and ROOS samples (938.53 ± 195.71 pg/ml; *p* = 0.005; Figure 4H).

In contrast, *IL8* expression of NaIO_3_ (0.38-fold expression), BSA (0.65-fold expression), HPR (0.57-fold expression), as well as ROOS ppRPE cells (0.45-fold expression) was not altered (all: *p* > 0.050; Figure 4I).

In addition, ELISA measurements were compared for each group between 16 and 24 days. Regarding IL-6, the levels were significantly higher in CTRL supernatants at day 24 compared to day 16 (*p* = 0.002), while no changes were noted in the NaIO_3_ group at both points in time (*p* = 0.088). Furthermore, IL-6 levels were significantly higher at day 24 when compared to day 16 in BSA (*p* = 0.004), HPR (*p* = 0.002), and ROOS samples (*p* = 0.008; Figure 4J).

The IL-8 levels were not altered when comparing day 24 to day 16 in CTRL samples (*p* = 0.647). In NaIO_3_ supernatants, higher IL-8 levels were noted at day 24 when compared to day 16 (*p* < 0.001). No changes were observed in the BSA group at both points in time (*p* > 0.050), while the IL-8 level was decreased at day 24 compared to day 16 in HPR samples (*p* < 0.001). In ROOS supernatants, the IL-8 levels did not differ between day 16 and day 24 (*p* > 0.050; Figure 4K).

### 3.6 NaIO_3_ supplemented media promotes oxidative stress in ppRPE cells

**FIGURE 5:**
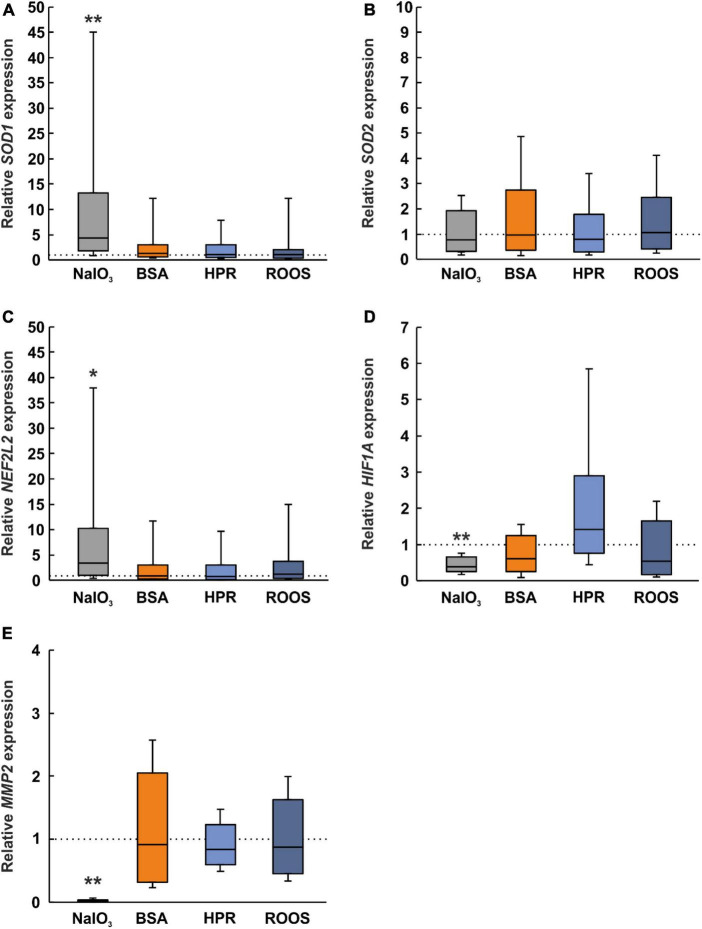
Altered expression of oxidative stress, hypoxia, and ECM marker in NaIO_3_ samples. **(A)** A marker for oxidative stress, *SOD1*, was upregulated in the NaIO_3_ group, while the other supplemented groups (BSA, HPR, and ROOS) showed comparable results as CTRL. **(B)** Interestingly, *SOD2* was not regulated in any group. **(C)** Expression of *NFE2L2* was elevated in the NaIO_3_ group compared to CTRL. **(D)** *HIF1A*, a marker for hypoxia, was downregulated in the NaIO_3_ group compared to CTRL. **(E)** *MMP2* is involved in extracellular matrix remodeling and was significantly decreased in the NaIO_3_ group when compared to CTRLs. BSA, bovine serum albumin; HPR, homogenized porcine retina; ROOS, rod outer segments. Values are median ± quartile ± minimum/maximum, the dotted lines represent the expression levels of the CTRL group, *n* = 6/group, *p < 0.050, **p < 0.010 vs. CTRL.

Previous studies suggested a key role of oxidative stress in the pathogenesis of AMD. Since oxidative stress is involved in almost all suspected risk factors of AMD, it might be crucial for the development and progression of this disease (Datta et al., 2017). An increase in superoxide dismutase (SOD) is seen as an indicator of oxidative stress (Liu et al., 2022). The *SOD1* expression was significantly upregulated in NaIO_3_ exposed ppRPE cells (4.38-fold expression; *p* = 0.005) in contrast to CTRL ppRPE cells. BSA (1.35-fold expression), HPR (1.08-fold expression), and ROOS (1.09-fold expression) samples showed a comparable *SOD1* mRNA expression as CTRLs (all: *p* > 0.050; Figure 5A).

On the other hand, *SOD2* expression was comparable within all groups (NaIO_3_: 0.77-fold expression, BSA: 0.96-fold expression, HPR: 0.80-fold expression, ROOS: 1.06-fold expression; all: *p* > 0.050; Figure 5B).

According to the literature, nuclear factor erythroid 2-related factor 2 (NFE2L2) may regulate the expression of antioxidant proteins. This protein could protect against oxidative damage caused by injury and inflammation (Bellezza, 2018). On the one hand, in ppRPE cells exposed to NaIO_3_, the *NFE2L2* expression was significantly enhanced in contrast to CTRL ppRPE cells (3.32-fold expression; *p* ≤ 0.050). On the other hand, the *NFE2L2* expression was unaltered in BSA (0.86-fold expression), HPR (0.71-fold expression), as well as in ROOS ppRPE cells (1.19-fold expression; all: *p* > 0.050; Figure 5C).

Besides oxidative stress, hypoxia is often discussed in context with deposits and AMD pathogenesis. *HIF1A* is linked with choroidal neovascularization and appears to be a key transcription factor in retinal angiogenesis. The expression of *HIF1A* was significantly downregulated in the NaIO_3_ group (0.39-fold expression; *p* = 0.002). No changes were noticed in the other groups (BSA: 0.61-fold expression, HPR: 1.42-fold expression, ROOS: 0.54-fold expression; all: *p* > 0.050; Figure 5D). Hence, our results indicate that oxidative stress, like hypoxia, is only effect by using NaIO_3_ supplemented media on ppRPE cells.

### 3.7 Extracellular matrix remodeling seems to be not triggered by the application of deposit media

Dysregulation of the ECM by altering matrix metalloproteinases (MMPs) has also been associated with increased risk of AMD (Zarbin, 2004; Zeng et al., 2013). The expression of *MMP2* was evaluated and a significant decrease was found in NaIO_3_ exposed ppRPE cells in comparison to the control group (0.02-fold expression; *p* = 0.002). No alterations were seen for BSA (0.91-fold expression), HPR (0.84-fold expression), or ROOS samples (0.88-fold expression; all: *p* > 0.050; Figure 5E).

### 3.8 Supplemented media diminished cone cells in cocultured neuroretina samples

**FIGURE 6:**
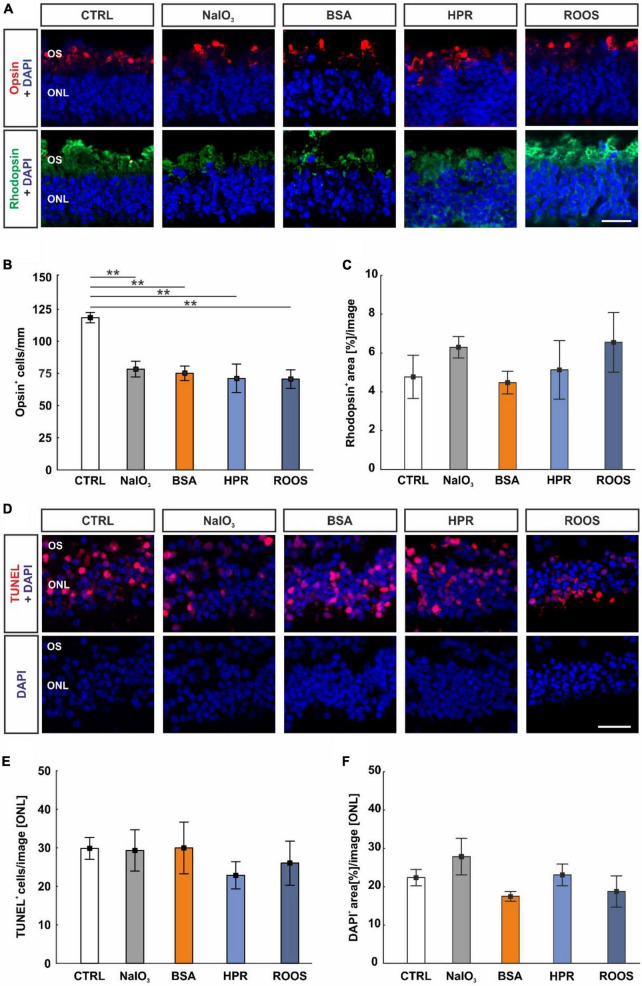
Supplemented media led to decreased cone cell numbers. **(A)** In neuroretina cross-sections, cones were marked with M/L-opsin (red) and rods with rhodopsin (green). Cell nuclei were stained with DAPI (blue). **(B)** The number of M/L-opsin^+^ cells was significantly reduced in all four supplemented groups in comparison to CTRLs. **(C)** The rhodopsin^+^ signal area was comparable in all groups. **(D)** Apoptotic cells were labeled with TUNEL (red), while DAPI (blue) counterstained cell nuclei. **(E)** The number of TUNEL^+^ cells within the ONL was constant in all groups. **(F)** The DAPI^+^ area in the ONL was similar in all groups. CTRL, control; BSA, bovine serum albumin; HPR, homogenized porcine retina; ROOS, rod outer segments; ONL, outer nuclear layer; OS, outer segments. Scale bars: 20 μm, values are mean ± SEM, *n* = 4–5/group, **p < 0.010 vs. CTRL.

Since photoreceptor degeneration is a hallmark of AMD, these cell types were studied in neuroretina samples cocultured with ppRPE cells that were exposed to supplemented media beforehand. To assess the maintenance of M/L-cones and rods, cross-sections of the neuroretina of all groups were immunohistochemically stained via M/L-opsin as well as rhodopsin and evaluated. The opsin^+^ cells appeared organized in rows located within the outer photoreceptor segment (OS; Figure 6A). The number of M/L-cones was significantly reduced in all supplemented medium groups (NaIO_3_: 75.25 ± 5.61 cells/mm, BSA: 78.46 ± 6.16 cells/mm, HPR: 71.20 ± 11.07 cells/mm, ROOS: 70.64 ± 7.20 cells/mm) in contrast to CTRL tissue (118.49 ± 3.97 cells/mm; all: *p* < 0.010; Figure 6B).

In contrast, rhodopsin staining revealed no differences in the staining area of the neuroretina samples within all groups (CTRL: 4.77 ± 1.11 area[%]/image, NaIO_3_: 6.29 ± 0.55 area[%]/image, BSA: 4.47 ± 0.58 area[%]/image, HPR: 5.13 ± 1.51 area[%]/image, ROOS: 6.55 ± 1.54 area[%]/image; all: *p* > 0.050; Figure 6C).

To detect a specific effect on cone and rod survival, a TUNEL assay was performed (Figure 6D). Interestingly, the number of TUNEL^+^ cells in the ONL was equal in all groups (CTRL: 29.87 ± 2.83 cells/image, NaIO_3_: 29.98 ± 6.69 cells/image, BSA: 29.32 ± 5.34 cells/image, HPR: 22.89 ± 3.52 cells/image, ROOS: 26.03 ± 5.72 cells/image; all: *p* > 0.050; Figure 6E).

A possible loss of cells in the ONL was evaluated by area analyses of DAPI cells (Figure 6D). Here, DAPI^+^ area was similar within all group (CTRL: 22.38 ± 2.13 area[%]/image, NaIO_3_: 27.87 ± 4.76 area[%]/image, BSA: 17.48 ± 1.26 area[%]/image, HPR: 23.09 ± 2.82 area[%]/image, ROOS: 18.77 ± 4.10 area[%]/image; all: *p* > 0.050; Figure 6F).

### 3.9 Interleukin-8 is altered in cocultured neuroretina samples

**FIGURE 7:**
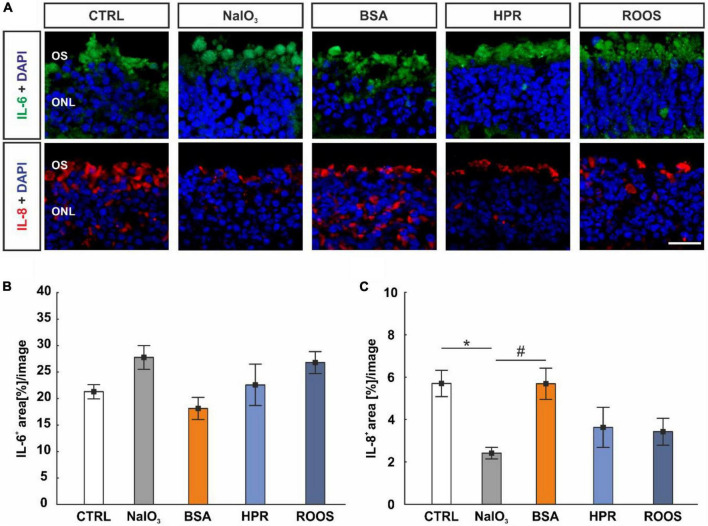
IL-8 diminished in NaIO_3_ neuroretinae. **(A)** Neuroretinal cross-sections were labeled with antibodies against IL-6 (green) and IL-8 (red), while DAPI counterstained cell nuclei (blue). **(B)** The IL-6^+^ staining area did not differ within the groups. **(C)** IL-8 staining revealed a smaller staining area in NaIO_3_ samples compared to CTRLs. Compared to NaIO_3_ supplemented neuroretinae, a higher IL-8^+^ area was noted in BSA specimens, while no difference was noted compared to CTRLs. CTRL, control; BSA, bovine serum albumin; HPR, homogenized porcine retina; ROOS, rod outer segments; ONL, outer nuclear layer; OS, outer segments. Scale bar: 20 μm, values are mean ± SEM, *n* = 4–5/group, *p < 0.050 vs. CTRL, ^#^*p* < 0.050 vs. NaIO_3_.

An anti-IL-6 antibody was used to label this cytokine on neuroretina sections of all groups (Figure 7A). Here, the evaluation showed no alterations in the IL-6^+^ staining area in the ONL and within the samples (CTRL: 21.30 ± 1.35 area[%]/image, NaIO_3_: 27.77 ± 2.23 area[%]/image, BSA: 18.12 ± 2.09 area[%]/image, HPR: 22.58 ± 3.90 area[%]/image, ROOS: 26.79 ± 2.07 area[%]/image; all: *p* > 0.050; Figure 7B).

In addition, IL-8 was marked with an anti-IL-8 antibody on retinal cross-sections (Figure 7A). In NaIO_3_ samples (2.42 ± 0.27 area[%]/image), the IL-8^+^ staining area was significantly diminished compared to CTRL (5.71 ± 0.62 area[%]/image; *p* = 0.036). While no difference was noted in BSA neuroretinae (5.70 ± 0.74 area[%]/image) when compared to CTRL (*p* > 0.050), the IL-8^+^ staining area was larger compared to NaIO_3_ samples (*p* = 0.037). The staining area of IL-8 did not differ in HPR (3.63 ± 0.53 area[%]/image) and ROOS specimens (3.43 ± 0.63 area[%]/image) compared to CTRL neuroretinae (*p* > 0.050; Figure 7C).

## 4 Discussion

AMD is a very complex disease involving many factors such as inflammation, oxidative stress, hypoxia, and ECM remodeling (Shen et al., 2007; Parmeggiani et al., 2012; Fernandez-Godino et al., 2016; Armento et al., 2021). So far, there is a lack of adequate models that cover a broad spectrum of these mechanisms and allow their investigation. Our cocultivation model aims to facilitate the investigation of described mechanisms regarding deposit formation. The application of three different media supplements, BSA, HPR, and ROOS, to ppRPE cells should potentially stimulate deposits, as previously demonstrated by Amin et al. (2004). NaIO_3_ was used to mimic oxidative stress and cause degeneration of ppRPE cells as a degenerative control (Zhang et al., 2016; Liu et al., 2022). Subsequently, the effect and potential of the media to induce debris was evaluated with respect to the different AMD-associated pathomechanisms. Moreover, as a novel approach we combined these pretreated ppRPE with a neuroretina explant to investigate whether cocultivation affects the neuronal integrity and viability.

After isolation and cultivation, including eight days of exposure to different supplemented media and a subsequent cocultivation phase with a neuroretina explant, the ppRPE cells still displayed characteristic cobblestone RPE morphology and dark pigmentation. This shows that these properties are not affected by the different supplemented media. Moreover, the brightfield images suggested a first impression of an adverse effect of the media. This indicates a cytotoxic potential of the supplemented media in comparison to the ppRPE medium of the CTRL group. In our study, PI staining was performed to assess degenerating aspects via the application of NaIO_3_, BSA, HPR, or ROOS in comparison to the CTRL situation. All supplemented media resulted in the loss of ppRPE cells, as evidenced by a significantly reduced total cell number in culture. In particular, the application of NaIO_3_ led to a severe reduction in the total number of ppRPE cells and a relatively strong increase of dead cells. Accordingly, Zhao et al. (2011) demonstrated that adding non-lethal doses of NaIO_3_ led to RPE cell dysfunction. In ARPE-19 cells, NaIO_3_ treatment induced cell death via ferroptosis (Liu et al., 2021). Huang et al. (2009) made quite similar observations, when treating human RPE cells with different BSA media. Increased intracellular formation of reactive oxygen species was observed, which was accompanied by exaggerated RPE cell death (Huang et al., 2009). Accordingly, human RPE cells exposed to ROOS showed mitochondrial DNA damage and apoptosis, evaluated in a cell culture model (Jin et al., 2001). In summary, exposure to NaIO_3_, BSA, ROOS, and HPR media has deleterious effects on the RPE cells, with NaIO_3_ representing the most cytotoxic potential.

RPE65 is expressed in the RPE and is essential during its development (Hamel et al., 1993). It is assumed to correlate with the expression of photoreceptor-specific proteins (Manes et al., 1998). RPE65 is pivotal for the visual cycle, which is necessary for both rod and cone vision (Redmond et al., 2005; Cai et al., 2009). In our study, RPE65 immunoreactivity was significantly diminished in all supplemented media groups in comparison to CTRL ppRPE cells, while mRNA expression of *RPE65* was significantly lower only in the NaIO_3_ and the BSA group. This degenerative effect of NaIO_3_ is in accordance with a study by Liu et al. (2022). The authors noticed an initial decrease of *Rpe65* expression in a rodent retinal degeneration model (Huang et al., 2009). Interestingly, when local tissue repair by RPE cell regeneration was initiated, *Rpe65* expression was also found to be increased. Similar observations were made in an acute RPE degeneration model in adult rats (Kim et al., 2018). Moreover, reduced number of RPE cells and severe damage after NaIO_3_-induced oxidative stress have been previously described (Kannan and Hinton, 2014; Zhang et al., 2016).

ZO-1 belongs to the zonula occludens protein family and is a peripheral membrane phosphoprotein (Fanning et al., 1998). The ability of RPE cells to form the blood-retina barrier via those tight junction proteins is essential for visual function. Evaluating ZO-1^+^ areas in this study, a significant smaller area was only found in ppRPE cells exposed to NaIO_3_, whereas the mRNA expression of *TJP1* was unaltered in all groups. A dysfunction of ZO-1 in an epithelial monolayer promotes loss of barrier function (Sugrue and Zieske, 1997). Hence, this could indicate that the supplemented media, which are more connected to deposit-formation, have no influence on the barrier function of the ppRPE cells. This result seems unexpected since drusen formation has a significant impact on the barrier function of RPE cells in AMD patients (Farjood and Vargis, 2017). Nonetheless, Hirata et al. (2014) showed that in ARPE-19 cells exposed to oxidative stress by adding H_2_O_2_, the expression of ZO-1 or the adherents-junction protein N-cadherin was also unaffected. On the other hand, claudin-1 was downregulated in this study (Hirata et al., 2014), which is contrary to our data that revealed a significantly smaller ZO-1^+^ area in the NaIO_3_ treated ppRPE cells, but no differences in the *TJP1* mRNA expression in all groups.

RLBP1 is a marker of visual cycle in RPE cells. In accordance with Tang et al. (2021) we observed a significant reduction of *RLBP1* expression in NaIO_3_ exposed ppRPE cells in contrast to CTRL ppRPE cells. Tang et al. (2021) noted reduced protein levels of RLBP1 and RPE65 in *in vivo* monolayer of mouse RPE cells from NaIO_3_ damaged retinae.

Variants of ApoE are closely linked with an increased risk of AMD. Besides, it is a major key component in lipoprotein metabolism and shows a decisive role in neuronal response to damage (Mahley and Rall, 2000). Moreover, ApoE itself can be a drusen component. Klaver et al. (1998) described a direct involvement of ApoE in the pathogenesis of AMD by demonstrating a presence of ApoE in disease-associated deposits in macular specimens of patients. Amin et al. (2004) detected sub-RPE deposits *in vitro* using ApoE immunoreactivity after exposing cells to media like ours. Therefore, we examined the expression of *APOE* mRNA in our cells. Interestingly, the HPR group displayed a significant upregulation of *APOE* expression in comparison to CTRLs, whereas the NaIO_3_ group showed significant downregulation. Accordingly, Amin et al. (2004) described an increased thickness of all types of characterized deposits in RPE cells exposed to HPR medium. In their study, they also documented a significant increase of deposits in ARPE-19 cells after exposing them to media supplemented with retinal homogenate. In our case, a significant upregulation of *APOE* mRNA in ROOS group was not detectable. Nevertheless, they monitored the deposit formation over longer study periods of five, seven, and eleven weeks, whereas our culture was only 24 days old (Amin et al., 2004). On this basis, it can be assumed that the HPR medium has a high potential to trigger deposit formation in our *ex vivo* ppRPE coculture model and should therefore be further analyzed at later points in time in the future. Furthermore, in our study, NaIO_3_ also had an influence on *APOE* expression, although there was a downregulation. A study on a radial glial cell line (L2.3) showed an NaIO_3_-induced inhibition of the Wnt/β-catenin signaling (Chen et al., 2014). Defective WNT signaling could in turn be linked to ApoE (Palomer et al., 2019). Consequently, NaIO_3_ could exert its toxic effect in ppRPE cells via this signaling pathway, which requires further analysis.

As mentioned, the NLRP3 inflammasome is linked to AMD (Doyle et al., 2012; Kauppinen et al., 2012; Tarallo et al., 2012; Tseng et al., 2013). Both drusen as well as their components, such as the complement protein C1q, can activate the NLRP3 inflammasome. Doyle et al. (2012) documented that isolated human drusen samples exhibit *NLRP3* expression. Therefore, they hypothesized that NLRP3 acts as a sensor leading to inflammasome activation (Doyle et al., 2012). In addition, evidence was found that the NLRP3 inflammasome is present in both, RPE and adjacent drusen, during the pathogenesis of advanced stages of AMD (Celkova et al., 2015). In this context, we also investigated whether exposure to the various supplemented media could trigger inflammatory reactions and exert cytotoxic effects involving the NLRP3 inflammasome. However, only the NaIO_3_ group showed a significantly increased *NLRP3* expression. This implies that the media thought to trigger the formation of deposits in ppRPE cells had no effect on the formation of an NLRP3 inflammasome in our study. The exact function of NLRP3 is still controversial in the literature. Activation of the inflammasome leads to the production of IL-18, which has been described as both harmful (Tarallo et al., 2012) and protective (Doyle et al., 2012), so the function of NLRP3 in regard to drusen formation is not fully understood yet. Kauppinen et al. (2012) discovered that oxidative stress in ARPE-19 cells leads to an upregulation of *NLRP3* expression. Since oxidative stress in our coculture model also only occurred in the NaIO_3_ group, this could explain the lack of inflammasome activation by the other supplemented media (BSA, HPR, ROOS).

Hageman et al. (2001) noted an inflammatory immune response in AMD in association to deposit formation. RPE cells secrete inflammatory mediators, such as TNF-α, as well as cytokines, like IL-6 and IL-8 (Leung et al., 2009). Notably, all supplemented media in our study led to a significant upregulation of *TNF* expression. TNF-α is a pro-inflammatory cytokine released by macrophages and T-cells in retina. Studies have shown that TNF-α is an important regulator of photoreceptor autophagy after retinal detachment (Xie et al., 2017). Therefore, TNF-α levels are closely related to photoreceptor cell homeostasis. Also, local dysregulation of immune mediators is involved in AMD pathogenesis. Thus, the increased *TNF* expression in the NaIO_3_, the BSA, the HPR, and the ROOS group might have an impact on photoreceptor viability in the cocultured neuroretina. A dysfunctional RPE and degenerated photoreceptors plays a central role in the pathobiology of AMD. Mimicking this in our coculture system would be a critical step towards an *ex vivo* AMD model.

Ma et al. (2009) analyzed the role of activated retinal microglia *in vitro* by coculturing them with primary RPE cells to assess their involvement in RPE dysfunction with respect to the pathological features described in AMD. They demonstrated microglia-RPE interactions that led to changes in RPE structure as well as distribution of microglia and promoted secretion of proinflammatory molecules. Taken together, these pathogenic aspects induce a microenvironment that triggers the progression of AMD pathogenesis (Ma et al., 2009). To evaluate the involvement and function microglia in our models, we examined *ITGAM* expression in ppRPE after the exposure to our four supplemented media. Interestingly, we found a significant downregulation of *ITGAM* in the BSA and NaIO_3_ group in comparison to the CTRL group. Microglia function as resident immune cells of the retina. However, their functions are divers, they are able to secrete cytokines, chemokines, neurotrophic factors, and neurotransmitters, which, in turn, can lead to cytotoxic but also cytoprotective effects (Hanisch and Kettenmann, 2007). Thus, the observed various expression of *ITGAM* in response to different media exposure might be quite interesting. Ma et al. (2009) reported that the influence of activated microglia leads to significant changes in various properties and gene expression of RPE cells. The application of NaIO_3_ in animal as well as in *in vitro* models led to a degeneration due to increased oxidative stress (Kannan and Hinton, 2014; Wang et al., 2014; Hanus et al., 2016; Berkowitz et al., 2017; Liu et al., 2021; Yang et al., 2021). Hence, it can be assumed that the reduction of *ITGAM* expression is a reaction of the degradation of ppRPE cells. Interestingly, supplementation of BSA to the medium also led to reduced *ITGAM* expression, although the mechanism responsible for this still needs to be clarified.

As mentioned, cytokines like IL-6 or IL-8 are released in inflammatory response. Strikingly, the highest IL-6 level was detected in supernatant from the ROOS group after 16 days. However, the IL-6 level in supernatant of ppRPE cells of the NaIO_3_ group was barely detectable and thus significantly reduced compared to the ROOS group. Furthermore, there was a significantly elevated IL-6 level in supernatant from the BSA, HPR and ROOS groups compared to CTRL supernatant. At 16 days, a significantly lower IL-8 level was measured in supernatants of the NaIO_3_ group. In addition, the IL-8 level in the HPR group was significantly higher than in the CTRL group. Like the IL-6 levels, the IL-8 concentration in the supernatant of NaIO_3_ samples was significantly lower than in all other groups at the 24-day time point. Interestingly, both cytokines seem to be differentially regulated and secreted, as a significant increase of *IL6* mRNA expression was detectable after NaIO_3_ induced damage, whereas *IL8* expression remained unchanged in all cultivation conditions. Increased *IL6* expression has been observed after intravenous injection of NaIO_3_ in adult mice before. Moreover, RPE cell damage was found to be related to increased inflammatory reaction and macrophage activity (Moriguchi et al., 2018). The secretion of both pro-inflammatory cytokines, IL-6 and IL-8, is associated with AMD onset and progression (Seddon et al., 2005; Goverdhan et al., 2008; Tsai et al., 2008; Ricci et al., 2013; Klein et al., 2014). Dib et al. (2015) detected more IL-6 and IL-8 secretion triggered by mitochondrial DNA in ARPE-19 cells. They discovered that the extent of expression was dependent on the amount of mitochondrial DNA (Dib et al., 2015). Therefore, the higher IL-8 concentration in the HPR group at day 16 in our study could be hint toward a potential induction of deposits in this coculture. Goverdhan et al. (2008) described a polymorphism in IL-8 promoter gene as a potential risk factor for AMD since they could detect this polymorphism more frequently in AMD patients. Nevertheless, the different regulation of IL-6 and IL-8 should be further investigated.

Inflammation and oxidative stress play an important role in the development of AMD by promoting the degeneration of the RPE (Imamura et al., 2006; Shen et al., 2007; Hollyfield et al., 2008; Shaw et al., 2012; Shadrach et al., 2013; Aredo et al., 2015; Rohrer et al., 2016). Thus, besides inflammatory processes, we also wanted to investigate the involvement of oxidative stress in response to the different media in our *in vitro* model. In particular, the addition of NaIO_3_ has been used in many *in vivo* and *ex vivo* studies to investigate oxidative stress in association with AMD related pathological changes (Kiuchi et al., 2002; Carido et al., 2014; Kannan and Hinton, 2014; Moriguchi et al., 2018; Enzbrenner et al., 2021). The activities of antioxidant enzymes SOD1 and SOD2 in RPE cells serve as indicators of oxidative stress. Interestingly, in our set - up, *SOD1* expression was significantly increased in the NaIO_3_ group, while no differences were noted in the other supplemented groups.

Interestingly, the expression of *NFE2L2*, a transcription factor for cell protection, was comparable to the CTRL group regarding BSA, HPR, as well as ROOS samples, while NaIO_3_ samples exhibited significantly enhanced *NFE2L2* mRNA expression. Sachdeva et al. (2014) undermined the importance of Nrf2 pathway activation in response to oxidative stress, when examining young (2 months) and old (15 months) murine RPE cells after systemic administration of NaIO_3_ in an *in vivo* model. Interestingly, they noted increased expression of *Nfe2l2* in response to oxidative stress but unaltered levels in older mice (Sachdeva et al., 2014). Zhao et al. (2011) showed that NRF2-deficient mice are suitable as a model for AMD disease because the animals develop ocular pathology that has many comparable major features of human AMD. They concluded that deregulated autophagy probably signifies the mechanistic link between oxidative damage and inflammation (Zhao et al., 2011). Appropriately, Felszeghy et al. (2019) noted that NFE2L2 also has an important function in RPE cell structure and function, which is similar to AMD degeneration. They documented photoreceptor dysmorphology and vision loss in a double knockout NRF-2/PGC mouse model. This mimics aspects of AMD pathology seen in patients (Felszeghy et al., 2019). Our data suggest that a response occurs in ppRPE cells treated with NaIO_3_, leading to increased *NFE2L2* expression which may represent a protective mechanism against the induced oxidative stress.

Alterations in the ECM occur naturally in the eye during aging. However, they can become pathologic, leading to a reduction in function and ultimately to the accumulation of debris or deposits as well as drusen, a hallmark of AMD. Thus, an age-dependent dysfunction of RPE cells and linked dysregulation of ECM remodeling is associated with drusen formation. In this regard, MMPs are important for the regulation of the ECM (Vu and Werb, 2000; Page-McCaw et al., 2007). Interestingly, in our study, changes in *MMP2* expression were not detectable in the BSA, the HPR, or the ROOS group compared to CTRLs. In contrast, the expression of *MMP2* was significantly downregulated in the NaIO_3_ group. Hussain et al. (2011) detected a reduction in activated MMP2 levels in AMD donor samples. The authors hypothesized that this leads to impaired matrix degradation of Bruch’s membrane. This, in turn, leads to AMD associated pathology (Hussain et al., 2011), suggesting that NaIO_3_ treatment induces AMD-like mechanisms.

In summary, using different supplemented media (NaIO_3_, BSA, HPR, and ROOS), the ppRPE cells were exposed to inflammation, oxidative stress, and degeneration. NaIO_3_ had the most prominent effects in the current study. The concentration and incubation of the media supplements certainly play a crucial role in the noted effect.

Previous studies observed that coculturing RPE cells and neuroretina has a beneficial effect on photoreceptor cell maintenance *in vitro* (Kaempf et al., 2008; Di Lauro et al., 2016; Mollick et al., 2016; Wagner et al., 2022a,b). In our study, RPE cells were exposed to various supplemented media (NaIO_3_, BSA, HPR, ROOS) and then cocultured with neuroretina. We observed a cytotoxic effect on the ppRPE cells. This suggests that as the number of ppRPE cells drops, the preservative effect on the neuroretina also decreases. This is reflected in a significant reduction in the number of M/L-opsin^+^ cells in the supplemented groups compared to CTRLs. Surprisingly, however, this effect does not seem to have a major impact on rod conservation. Rhodopsin^+^ area was comparable in all groups. Especially, due to the close localization and interaction of the two photoreceptor cells (Fain and Sampath, 2018), this different response to the degeneration of the ppRPE cells is surprising. However, the group difference seems to be small as the number of TUNEL^+^ cells as well as the DAPI^+^ area in the ONL were similar in all groups. In addition, the different evaluation procedures for quantification of opsin and rhodopsin might explain the discrepancies. Furthermore, we evaluated the response of IL-6 and IL-8 in cocultured neuroretinae. While no alterations were observed for IL-6, a smaller IL-8 staining area was noted in NaIO_3_ samples. Similar results were seen in human RPE cells, treated with different dose of NaIO_3_. Here, the authors noted a decrease in IL-8 expression through ELISA measurements from RPE cells (Zhang et al., 2016). It needs to be kept in mind that immunohistological stainings are only semi-quantitative assessments. Therefore, in further studies, photoreceptors and interleukins need to be examined in more detail.

It should be mentioned that the study presented here has some limitations. We performed RT-qPCR evaluations for several markers that point to different characteristics associated with AMD in our *ex vivo* model. These findings should be verified with techniques like bulk mRNA seq analysis or verified via e.g., Western blot analysis, on protein levels in follow-up studies. In this first study using ppRPE cells, we cultivated these cells with supplemented media. Then, these cells were cocultivated with porcine neuroretina to mimic the disease pattern of AMD. All supplemented media had damaging effects on ppRPE cells, resulting in cell loss. Some hints for deposits were noted, a significant upregulation of APOE was seen in HPR ppRPE cells. Hence, this finding should be investigated in more detail in future studies.

## 5 Conclusion

All supplemented media in this study, namely NaIO_3_, BSA, HPR, and ROOS, resulted in decreased expression of the RPE characteristic proteins RLBP1 and RPE65. Our data suggest that the supplemented media have an adverse effect on exposed ppRPE cells compared to CTRL medium. Our hypothesis that certain supplements induce the formation of deposits in RPE cells could not be fully confirmed. Only the HPR group showed some potential. Here, an upregulation of the drusen marker *APOE* could be observed. All supplemented media had a negative impact on the cone cells of cocultured neuroretina. In addition, several aspects such as inflammation, oxidative stress, and ECM remodeling could be investigated in relation to the application of different media and potential deposits. Interestingly, none of these features appear to be associated with or modified by deposit formation in our model. In future studies, further experiments are planned to analyze the neuroretina explants in more detail. Furthermore, it has to be investigated whether the orientation of the explants with the photoreceptor layer upward represents a change in the interaction, which may well show an influence during coculture.

## Data availability statement

The original contributions presented in this study are included in this article/supplementary material, further inquiries can be directed to the corresponding author/s.

## Ethics statement

Ethical approval was not required for the study involving animals in accordance with the local legislation and institutional requirements.

## Author contributions

NW: Data curation, Formal analysis, Funding acquisition, Investigation, Visualization, Writing – original draft. TT: Formal analysis, Validation, Visualization, Writing – original draft. SR: Formal analysis, Validation, Visualization, Writing – original draft. JT: Data curation, Investigation, Writing – review & editing. HD: Resources, Writing – review & editing. SJ: Conceptualization, Funding acquisition, Project administration, Supervision, Writing – original draft.
